# Extended-Spectrum Beta-Lactamase-Producing *Enterobacteriaceae* in Critically Ill Patients: Impact of Carriage And Infection On Carbapenem Consumption, Duration of Icu Stay, And Mortality

**DOI:** 10.1186/2197-425X-3-S1-A1

**Published:** 2015-10-01

**Authors:** F Barbier, C Pommier, M Garrouste-Orgeas, C Schwebel, S Ruckly, AS Dumenil, V Lemiale, B Mourvillier, C Clec'h, M Darmon, V Laurent, G Marcotte, B Souweine, JR Zahar, JF Timsit, for the OUTCOMEREA Study Group

**Affiliations:** La Source Hospital - CHR Orléans, Medical ICU, Orléans, France; Paris XI University, Paris, France; Saint-Joseph Hospital Network, Medical-Surgical ICU, Paris, France; Albert Michallon University Hospital, Medical ICU, Grenoble, France; Outcomerea, Department of Biostatistics, Bobigny, France; Antoine Béclère University Hospital, Surgical ICU, Clamart, France; Saint-Louis University Hospital, Medical ICU, Paris, France; Bichat-Claude Bernard University Hospital, Medical and Infectious Diseases ICU, Paris, France; Avicenne University Hospital, Medical ICU, Bobigny, France; Saint-Etienne University Hospital, Medical ICU, Saint-Priest en Jarez, France; André Mignot Hospital, Medical ICU, Versailles, France; Edouard Herriot University Hospital, Surgical ICU, Lyon, France; Gabriel Montpied University Hospital, Medical ICU, Clermont-Ferrand, France; Angers University Hospital, Infection Control Unit, Angers, France

## Introduction

The spread of extended-spectrum beta-lactamase-producing *Enterobacteriaceae* (ESBL-E) is a major issue in ICU patients. Whether ESBL-E carriage and infection impact the outcome and the use of carbapenems in this population remains scarcely evaluated.

## Objectives

We investigated the effects of ESBL-E carriage and infection on carbapenem consumption, ICU length of stay (LOS), and adjusted probability of death at day-28 (D28) in a multicenter cohort of critically ill patients.

## Methods

Adult patients admitted between January 1996 and December 2013 in 17 French ICUs contributing to the OUTCOMEREA prospective database were included. Screening for ESBL-E carriage was performed at ICU admission and weekly thereafter. a cause-specific hazard model was built to assess the impact of carriage with and without ICU-acquired infection due to ESBL-E on the likelihood of death or ICU discharge at D28.

## Results

Among the 16,734 included patients, 594 (3.5%) were colonized with ESBL-E during their ICU stay (imported carriage, 52.2%). a total of 118 ESBL-E infections occurred in 98 carriers (16.4%). Both ESBL-E infections and ESBL-E carriage without infection increased the use of carbapenems during the ICU stay when compared to non-carriage (627, 241 and 69 treatment days for 1,000 patient-days, respectively, p < 0.001 for all comparisons). The ICU LOS was longer in carriers than in non-carriers (median [IQR], 13 [6-26] vs 5 [3-9] days, p < 0.01) but similar in carriers with and without ESBL-E infection (13 [6-29] vs 13 [6-26] days, p = 0.52). Crude mortality rates at D28 were higher in carriers than in non-carriers (19.7% vs 15.6%, p < 0.01), and further differed when comparing carriers with ESBL-E infections and uninfected carriers (30.6% vs 17.5%, p < 0.01). After adjustment on baseline (chronic diseases, type of admission, and SAPS II and organ failures at ICU admission) and time-dependent (SOFA score at the time of ESBL-E acquisition, and end-of-life decisions) variables, ESBL-E infections increased the risk of death at D28 [adjusted cause-specific hazard ratio (aCSHR) 1.825, 95% confidence interval (95%CI) 1.235-2.699, p = 0.003] and the ICU LOS (aCSHR 0.563, 95%CI 0.432-0.733, p < 0.001). ESBL-E carriage without infection prolonged the ICU stay (aCSHR 0.623, 95%CI 0.553-0.702, p < 0.001), but had no effect on D28 mortality (aCSHR 0.906, 95%CI 0.722-1.136, p = 0.4). When analyzed separately, the effects of carriage and infection did not differ between ESBL-producing *Escherichia coli* (48.7% of carriage isolates) and other ESBL-E.

## Conclusions

In this large cohort of critically ill patients, ESBL-E infections increased carbapenem consumption, ICU LOS, and D28 mortality. Less than one fifth of carriers developed an ESBL-E infection; however, even ESBL-E carriage without infection increased carbapenem exposure and delayed discharge, thereby amplifying both the selective pressure and the colonization pressure in the ICU.

